# Effects of Bushen Yiyuan recipe on testosterone synthesis in Leydig cells of rats with exercise-induced low serum testosterone levels

**DOI:** 10.1080/13880209.2022.2110126

**Published:** 2022-09-05

**Authors:** Yirong Wang, Xiyang Peng, Zhihong Zhou, Changfa Tang, Wenfeng Liu

**Affiliations:** a Institute of Physical Education, Hunan Normal University, Changsha, China; b Hunan Sports Vocational College, Changsha, China

**Keywords:** High-intensity training, physical fatigue, testes, Chinese medicine, *Panax ginseng*, *Epimedii folium*, pharmacological effects

## Abstract

**Context:**

Bushen Yiyuan recipe (BYR) is an effective Chinese prescription with antifatigue and antioxidation effects.

**Objective:**

The effects of BYR on testosterone synthesis in rat Leydig cells with exercise-induced low serum testosterone levels (EILST) are assessed.

**Materials and methods:**

Thirty-two Sprague-Dawley rats were chronically trained for 6 weeks to establish an EILST model. EILST rats were divided into model (physiological saline), EFE (700 mg/kg ethanol extract of *Epimedii folium*, the dried leaves of *Epimedium brevicornu* Maxim [Berberidaceae]), and BYR groups (350 and 700 mg/kg) for 6 weeks. Expression of HMG-CoA, LDL-R, SR-BI, STAR and CYP11A1 were quantified by RT qPCR and Western blots.

**Results:**

Compared with the model group (115.52 ± 13.05 μg/dL; 67.83 ± 14.29; 0.32 ± 0.04; 0.33 ± 0.02; 0.38 ± 0.01), serum testosterone, testosterone/cortisol ratio, HMG-CoA, STAR and CYP11A1 relative protein expression significantly increased in low-dose BYR (210.60 ± 5.08 μg/dL; 119.38 ± 13.02; 0.47 ± 0.01; 0.46 ± 0.03; 0.46 ± 0.02), high-dose BYR (220.57 ± 14.71 μg/dL; 124.26 ± 14.79; 0.49 ± 0.02; 0.42 ± 0.03; 0.51 ± 0.02), and EFE groups (206.83 ± 5.54 μg/dL; 119.53 ± 25.04; 0.45 ± 0.02; 0.42 ± 0.02; 0.41 ± 0.02) (all *p* < 0.01, except for CYP11A1 in EFE group). HMG-CoA, STAR and CYP11A1 mRNA relative expression significantly increased in low-dose and high-dose BYR group compared to model group (all *p* < 0.01).

**Conclusions:**

BYR affects endogenous cholesterol synthesis and testosterone synthesis to prevent and treat EILST levels in rats. It can improve the body’s sports ability.

## Introduction

A long period of high-intensity training can cause exercise-induced low serum testosterone levels (EILST) and lead to the decline of physical quality (Barone et al. 2013). The main reason for this phenomenon is that the functions of many components in the hypothalamic–pituitary–gonadal axis (HPG axis) are inhibited (Kaprara and Huhtaniemi 2018; Iwasa et al. 2018). It has been reported that the decrease of testosterone in Leydig cells is caused by the abnormal expression of Luteinizing Hormone/Choriogonadotropin (LH/CG) receptor, P450_SCC_, β2-adrenergic receptor, and other members of the HPG axis after exercise stimulation (Lin et al. 2001; Ge et al. 2005). However, the effects of exercise training and nutrition intervention on the key enzymes of testosterone biosynthetic pathways are rarely reported. Leydig cells of testis contain abundant amounts of endoplasmic reticula and mitochondria, which are sites for the synthesis and secretion of testosterone (Chung et al. 2020). There are four key processes during the synthesis process of testosterone in testicular Leydig cells: (1) endogenous synthesis of cholesterol; (2) intake and reverse transport of exogenous cholesterol; (3) transfer of cholesterol to the inner membrane of the mitochondria; (4) conversion of cholesterol to testosterone. Hydroxy-3-methyl glutaryl coenzyme A reductase (HMG-CoA), low-density lipoprotein receptor (LDL-R), and high-density lipoprotein receptor (SR-BI), steroidogenic acute regulatory protein (STAR), cholesterol side-chain cleavage cytochrome P_450_ enzyme (P_450_scc or CYP11A1) are key enzymes in those processes respectively (Liu et al. 2013).

Bushen Yiyuan recipe (BYR) is an effective prescription of traditional Chinese medicine in clinical application, composed of more than 10 kinds of extracts, including ginsenoside from *Ginseng radix et rhizome* (the dried root and rhizome of *Panax ginseng* C. A. Meyer [Araliaceae]), icariin from *Epimedii folium* (the dried leaves of *Epimedium brevicornu* Maxim. [Berberidaceae]), *Lycium* polysaccharide from *Lycii fructus* (the dried and mature fruits of *Lycium barbarum* Linn. [Solanaceae]), aqueous extracts of *Ligustri lucidi fructus* (the dried and mature fruits of *Ligustrum lucidum* Ait. [Oleaceae]), aqueous extracts of *Eucommiae cortex* (the dried bark of *Eucommia ulmoides* Oliver [Eucommiaceae]), aqueous extracts of *Astragali radix* (the dried root of *Astragalus membranaceus* (Fisch.) Bge. var. *mongholicus* (Bge.) Hsiao [Leguminosae]), aqueous extracts of *Agrimoniae herba* (the dried overground part of *Agrimonia pilosa* Ledeb. [Rosaceae]), aqueous extracts of Dashenjincao (the dried whole plant of *Phlegmariurus carinatus* (Desv.) Ching [Huperziaceae]), aqueous extracts of *Selaginellae herba* (the dried whole plant of *Selaginella tamariscina* (Beauv.) Spring [Selagioellaceae]), aqueous extracts of *Ajugae herba* (the dried whole plant of Ajuga decumbens Thunb. [Lamiaceae]) (Liu et al. 2018; Wang et al. 2016). Ginsenoside, icariin, *Lycium* polysaccharide are the main ingredients of BYR. Animal experiments show that this recipe can enhance the aerobic and anaerobic endurance of swimming mice, and has the effects of anti-free radical damage, improving cell energy metabolism and improving the cellular immune function (Zhou et al. 2000, 2001). The clinical research of sports medicine also shows that BYR can keep the blood testosterone concentration relatively stable, reduce the blood cortisol and increase the haemoglobin (Hb) level during the exercise training of male rowers, to enhance the effect of physical fitness of athletes (He et al. 2004). Besides, it can relieve the related symptoms of sports fatigue of rowers, such as mental fatigue, limb weakness and short sense of breath during exercise (He et al. 2004). However, the effect of BYR on testicular interstitial fibrosis and its molecular mechanism are still unclear.

In this study, the EILST model was established based on a treadmill training program with progressively increasing exercise load for rats, the intervention treatment was conducted with the high and low doses of BYR, and the extract of *Epimedii folium*, an extract that can significantly alleviate the impact of high-intensity exercise (HIE) on serum testosterone (Zhou et al. 2013). Therefore, *Epimedii folium* extract was chose as the positive control drug in the study. The ultrastructure of rat testicular Leydig cells, mRNA and protein expression of HMG-CoA, LDL-R SR-BI, STAR, and CYP11A1 were analyzed to explore the effectiveness of BYR on the prevention and treatment of EILST of rats and unveil the possible underlying molecular mechanisms.

## Materials and methods

### Experimental animals

Forty male SPF Sprague Dawley rats with the weight of 300 ± 10 g were provided by Hunan SJA Laboratory Animal Co., Ltd., and animal licence number is SCXK (Xiang) 2013-0004. The study protocol was approved (SCXK [Xiang] 2014-0002) by the Ethics Committee of Hunan Normal University (Changsha, China). Animal welfare and experimental procedures were approved by The University Committee on Animal Care of Hunan Normal University (SYXK [Xiang] 2014-0007) and carried out following the institutional guidelines of this committee and the care and use of laboratory animals (National Research Council 2010). We used pentobarbital sodium (50 mg/kg, intraperitoneal injection) as anaesthesia to minimize the suffering of rats.

### Reagents and materials

BYR was produced by Xi’an Acetar Bio-Tech Inc (Batch No.: 201500139) and was a brown mixed powder of ginsenoside (*Ginseng radix et rhizome* extract), icariin (*Epimedii folium* extract), *Lycium* polysaccharide (*Lycii fructus* extract), and other aqueous extracts of *Ligustri lucidi fructus*, *Eucommiae cortex*, *Astragali radix*, *Agrimoniae herba*, Dashenjincao, *Selaginellae herba*, and *Ajugae herba*. Each pill of BYR weighs 0.5431 g. Ginsenoside Rg1 (Lot No. 110703-201529, ≥95.0%), ginsenoside Re (Lot No. 110754-201626, ≥97.4%), ginsenoside Rb1 (Lot No. 110704-201424, ≥93.7%), ginsenoside Rb2 (Lot No. 111715-201203, ≥93.8%), Ginsenoside Rd (Lot No. 111818-201603, ≥92.1%), icariin (Lot No. 110737-201516, ≥94.2%) were purchased from the National Institute for the Control of Pharmaceutical and Biological Products (Beijing, China). Ginsenoside Rc (Lot No. Q3140025, ≥99.1%) was purchased from ANPEL Laboratory Technologies (Shanghai) Inc. (Shanghai, China). Trizol reagent (Cat. No. 15596026) and RT-PCR Kit (Cat. No. 10329) was purchased from Invitrogen Corporation (USA). DL2000 DNA Marker, Taq polymerase and dNTP were purchased from Genstar Technologies Co, Inc (USA). Tween-20 was purchased from Sigma Chemical Aldrich Co., Ltd. (St. Louis, MO, USA). RIPA buffer was purchased from Applygen Technologies Inc (Beijing, China). Rat-total cholesterol (Cat. No. A111-1-1), HDL-C (Cat. No. A112-1-1), LDL-C (Cat. No. A113-1-1) ELISA kits were purchased from Nanjing Jiancheng Bioengineering Institute (Nanjing, China). SDS-PAGE gel Kit (Cat. No. p1320) was purchased from Beijing Solarbio Science & Technology Co., Ltd. (Beijing, China). Polyvinylidene Fluoride membrane (Cat. No. BS-PVDF-22) was purchased from Beijing Labgic Technology Co., Ltd. (Beijing, China). Rabbit polyclonal anti-HMG-CoA (Cat. No. 13533-1-AP), anti-LDL-R (Cat. No. ab30532), anti-SR-BI (Cat. No. 21277-1-AP), anti-STAR (Cat. No. 12225-1-AP), anti-CYP11A1 (Cat. No. 13363-1-AP) were purchased from Proteintech Group (Wuhan, China). Antibodies against β-actin (Cat. No. 60008-1-Ig), secondary antibodies (Cat. No. HRP-60008) were provided by Proteintech Group (Wuhan, China). Super enhanced chemiluminescence (ECL) (Cat. No. 32209) was purchased from Thermo Fisher Pierce Co., Ltd. (USA).

### Groups and drug administration

Rats were randomly divided into 5 groups with 8 rats each including: normal group, model group, *Epimedii folium* extract (EFE) group (700 mg/kg), low-dose Bushen Yiyuan recipe (BYR) group (350 mg/kg), and high-dose BYR (700 mg/kg) The rats in the EFE group were daily treated with gavage EFE (700 mg/kg) (Wang et al. 2016); The low-dose BYR and high-dose BYR groups were daily treated with gavage BYR (350 or 700 mg/kg). The dosage of low-dose was equivalent to 6.3 times the human dose by intragastric administration (He et al. 2004; Song 2002). Drugs dissolved in physiological saline were administered by gavage in a total volume of 4 mL/kg/d. The EFE group, low-dose BYR group, and high-dose BYR group were administered intragastrically 1 h before exercise, once a day for 6 weeks, while the normal group and model group were fed normal diet and treated with physiological saline (4 mL/kg/d) only.

### Rat model of the exercise-induced low serum testosterone levels

The EILST rat model was established based on a treadmill training program with a progressively increasing load. The training program referred to the exercise training model established by Bedford et al. (1979) and was as described in Table 1, and the success of the rat model was based on the 15% decrease of testosterone content in the blood after a progressively increasing load aerobic training (Kumagai et al. 2018; Purvis et al. 2010). The training scheme for rats is listed in Table 1, in the first week, the animals were exercised at speed 15 m/min for 15 min on Day 1 and Day 2, at speed 15 m/min for 20 min on Day 3, at speed 18 m/min for 18 min on Day 4 and Day 5, at speed 18 m/min for 20 min on Day 6. All the rats had a rest on Sunday. All the exercise was performed on the treadmill with 0 grade.

**Table 1.** Training scheme for rats with exercise-induced low serum testosterone levels (meters/min × min).

	Day 1	Day 2	Day 3	Day 4	Day 5	Day 6	Day 7
1st week	15 × 15	15 × 15	15 × 20	18 × 18	18 × 18	18 × 20	Rest
2nd week	18 × 20	20 × 22	20 × 22	22 × 22	22 × 25	22 × 25	Rest
3rd week	22 × 25	25 × 28	25 × 28	25 × 28	27 × 30	27 × 30	Rest
4th week	27 × 30	29 × 32	29 × 32	29 × 35	29 × 35	29 × 35	Rest
5th week	29 × 38	29 × 38	29 × 38	29 × 40	29 × 40	29 × 40	Rest
6th week	29 × 40	29 × 40	29 × 40	29 × 40	29 × 40	Exhaustion	

### Effective ingredients determination of BYR by HPLC

HPLC analysis was performed on an Agilent 1260 system (Agilent Technologies, Santa Clara. CA, USA) equipped with a G1311C quaternary pump, a G1329B sampler, a G1316A column compartment, and a G4212B diode-array detector. Agilent 5 TC-C18 (4.6 mm × 250 mm, 5 μm) was used for HPLC analysis, the detection wavelengths were 203 nm and 270 nm, the column temperature was 30 °C, the flow rate was 1 mL/min. Mobile eluents were composed of aqueous solution (A) and acetonitrile (B). Gradient elution was used as follows: 24–24% B (76–76% A) at 0–20 min; 24–28% B (76–72% A) at 20–28 min; 28–30% B (72–70% A) at 28–30 min; 30–30% B (70–70% A) at 30–40 min; 30–40% B (70–60% A) at 40–42 min; 40–40% B (60–60% A) at 42–52 min; 40–24% B (60–76% A) at 52–62 min, consecutively. Appropriate amounts of ginsenoside Rg1, Re, Rb1, Rc, Rb2, Rd and icariin reference substance were weighed respectively, and mixed solutions, respectively, containing 0.3, 0.3, 0.3, 0.3, 0.3, 0.3 and 0.4 mg per 1 mL were prepared by adding methanol, and then shaken well. About 0.25 g of the BYR and EFE were respectively weighed, and then 10 mL of methanol was added into the conical flask, weighed and processed with ultrasound for 1 h. Then, supplemented with weight, shaken well and filtered. The mixed solution of reference substance, the extracts of BYR and herbal epimedii were absorbed 10 μL, respectively, and injected into HPLC for determination.

### Preparation of transmission electron microscopy (TEM) section samples

After 6 weeks of training, all rats were anaesthetized with 2% pentobarbital (50 mg/kg, intraperitoneal injection, i.p.) and decapitated. Under sterile conditions, right testicular tissues were taken out (testicular capsule was removed), washed, fixed, embedded, sliced and dyed according to the routine method (Baxa 2018) of electron microscopy, and transmission electron microscopy (TEM) (H7700, Hitachi, Japan) was used to observe the ultrastructure of rat Leydig cells.

### ELISA analysis and RT-qPCR analysis

Blood from rat abdominal aorta was collected, serum was separated and stored in the refrigerator at −20 °C. The levels of testosterone (T) and cortisol (C) in the serum were detected according to ELISA routine method (Weng and Zhao 2015) using ELISA microplate washer (PW-812, Huisong, China) and automatic ELISA microplate reader (MB-530, Huisong, China), and their absorbance values at the wavelength of 450 nm were recorded. The levels of total cholesterol (TC), high-density lipoprotein cholesterol (HDL-C), low-density lipoprotein cholesterol (LDL-C) in the serum were measured by ELISA kits following manufacturer’s instructions.

Leydig cells of testis were isolated and prepared by Percoll gradient centrifugation and frozen at −80 °C. The total RNA was extracted from the testis tissue of rats by Trizol reagent according to manufactures’ protocol (Shanghai Sangon; Code No. SK1322). Ultraviolet spectrophotometry was used to determine quantity of the extracted total RNA. RT-PCR kit (Shanghai Sangon; Code No. SK2442) was used for reverse transcription reactions. Aliquots of cDNA were amplified by fluorescent quantitative PCR machine (PIKO REAL 96, Thermo, USA), denaturation at 95 °C for 10 min, followed by 40 cycles of denaturation at 95 °C for 5 s and annealing at 60 °C for 30 s. For each amplification set, β-actin was run as the internal reference control. The 2^−ΔΔCT^ method was used to analyze the relative mRNA expression in the samples. The primer sequences are listed in Table 2.

**Table 2.** List of primer sequences for PCR.

Gene	Forward primer	Reverse primer
β-actin	5′-CATCCTGCGTCTGGACCTGG-3′	5′-TAATGTCACGCACGATTTCC-3′
HMG-CoA	CTGCGTGTCCCTGGTCCTA	TGGGTTACTGGGTTTGGTTTAT
LDL-R	GCCGCCTCTATTGGGTTGATT	TCTAGGCTGCGTGACGTTGTGA
SR-BI	TAGTCCTGCCATTGCTGTGGTT	CCTTATCCTGCGAGCCCTTTT
STAR	TGCCTGAGCAAAGCGGTGTC	CGCAAGTGGCTGGCGAACT
CYP11A1	TGAATGACCTGGTGCTTCGTAA	CCGGAAGTGCGTGGTGTTTT

### Western blot analysis of HMG-CoA, LDL-R, SR-BI, STAR, and CYP11A1 expression

Testicular tissue was removed from individual rats and homogenized in RIPA buffer. The homogenates were centrifuged at 12,000 *g* for 15 min, 20 mg of protein from the supernatants was then separated on 10% SDS-PAGE gel and transferred to BS-PVDF-22 membrane. Following the transfer, the PVDF membranes were blocked 2 h at room temperature with 5% skim milk in Tris-buffered saline (TBS, 20 mM Tris, 500 mM NaCl, pH 7.5), and then incubated with rabbit polyclonal anti-HMG-CoA (dilution ratio 1:1000), anti-LDL-R (dilution ratio 1:500), anti-SR-BI (dilution ratio 1:1000), anti-STAR (dilution ratio 1:1000), anti-CYP11A1 (dilution ratio 1:1000) in 1 × TBST overnight, at 4 °C. The membranes were washed 3 times for 15 min with Tris-buffered saline-Tween (TBST, 20 mM Tris, 500 mM NaCl, pH 7.5, 0.1% Tween 20). The membranes were then incubated with 1:3000 dilution anti-rabbit IgG (H + L) secondary antibodies in 1 × TBST for 50 min at room temperature. The membranes were washed three times for 15 min and the immunoproteins were detected by super ECL using hyper film and ECL reagent. The relative optical density of bands was quantified by densitometric scanning of the Western blot (Bio-Rad gel imaging system, USA).

### Statistical analysis

Data were expressed as the mean ± SD (*n* = 8), using SPSS for Windows (Version 20) for analysis. One-way analysis of variance (ANOVA) was used for comparison of differences between groups. LSD test was used for data with homogeneous variance and the Dunnett's T3 test used for data with uneven variance. *p* < 0.05 was considered as a significant difference, *p* < 0.01 was considered as a notable significant difference.

## Results

### Ginsenoside and icariin are major ingredients in BYR

**Figure 1.**
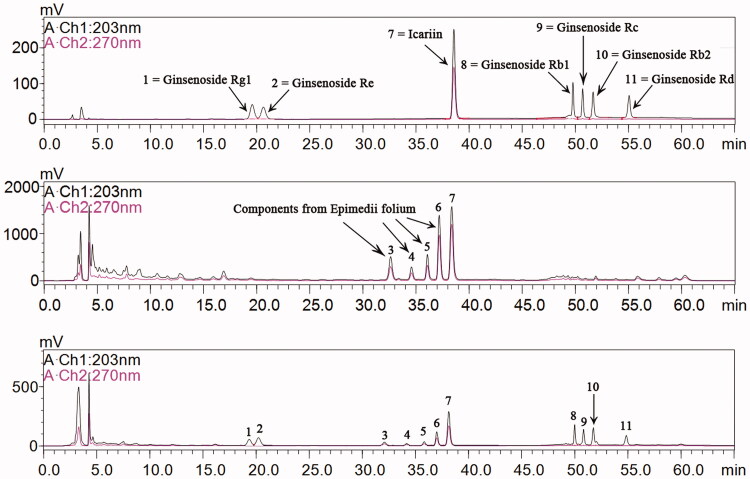
HPLC chromatogram analysis of (A) Mixed reference, (B) *Epimedii folium* extract, and (C) Bushen Yiyuan recipe. 1 = Ginsenoside Rg1, 2 = Ginsenoside Re, 3–6 = Components from *Epimedii folium*, 7 = Icariin, 8 = Ginsenoside Rb1, 9 = Ginsenoside Rc, 10 = Ginsenoside Rb2, 11 = Ginsenoside Rd with retention time 19.35, 20.23, 32.08, 34.17, 35.83, 37.01, 38.14, 49.99, 50.83, 51.75, and 54.85 min, respectively.

The major ingredients in BYR were analyzed by HPLC chromatogram. As shown in Figure 1, the chromatographic peaks 1, 2, 8, 9, 10 and 11 were characteristic peaks of ginsenoside. Therefore, according to the external standard method, each pill of BYR contained 43.57 mg of total ginsenoside. The chromatographic peak 3, 4, 5 6, and 7 were characteristic peaks of *Epimedii folium* extract (EFE), among which the chromatographic peak 7 represents icariin. Icariin has the largest peak area compared with the other four characteristic peaks. According to the external standard method, each pill of BYR contained 5.03 mg of icariin. The correlation coefficient was 0.9999, indicating the consistence of quality among the mixed standard and BYR extract, and demonstrating that BYR was produced consistently with good quality.

### BYR significantly increased serum testosterone levels but had no significant effect on serum cortisol, total cholesterol, HDL-C and LDL-C levels in EILST rats

**Figure 2.**
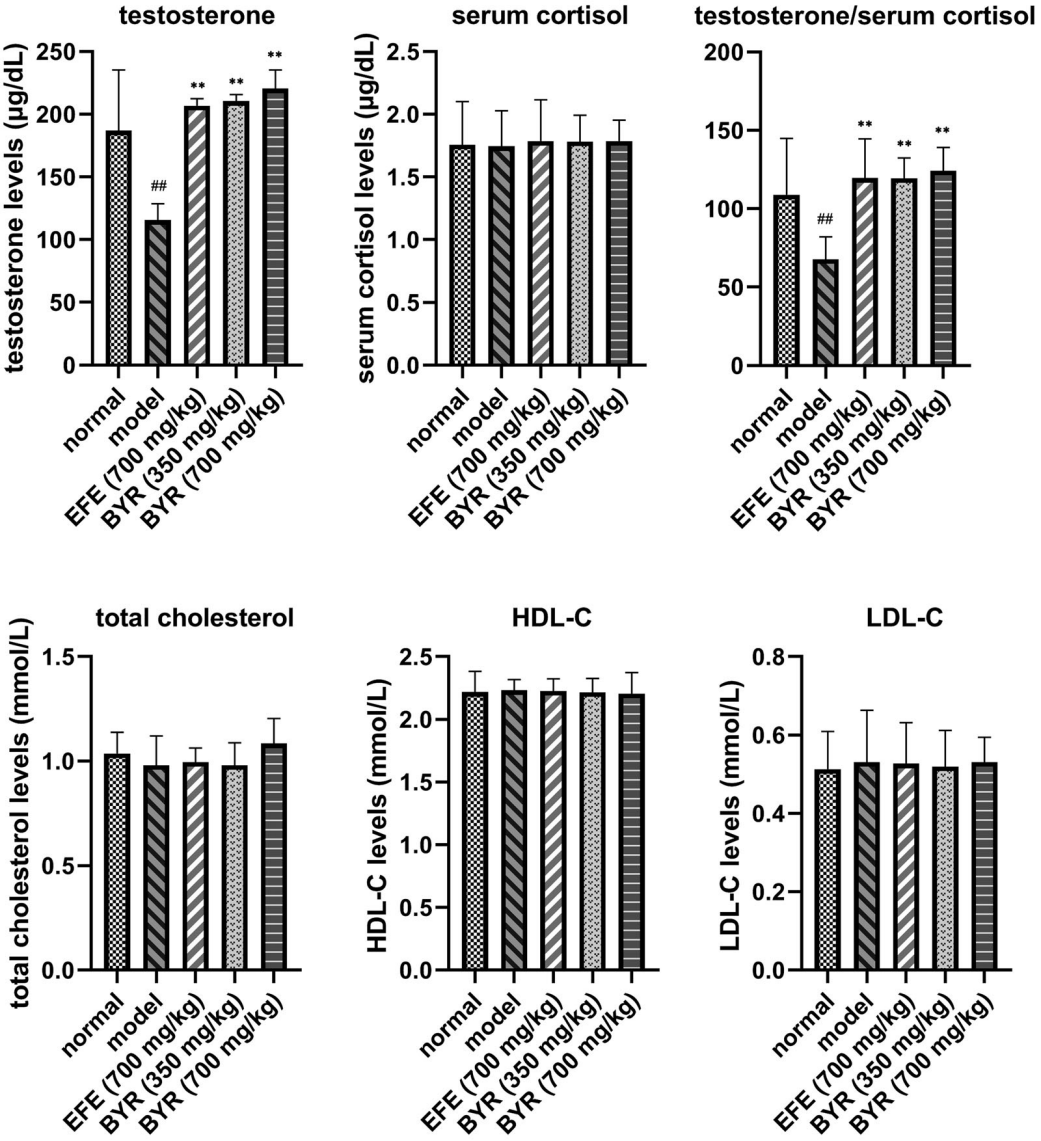
Testosterone, cortisol, testosterone/cortisol, total cholesterol, HDL-C and LDL-C levels in rats of normal group, model group, *Epimedii folium* extract (EFE) group, low-dose Bushen Yiyuan recipe (BYR) group and high-dose Bushen Yiyuan recipe (BYR) group. Data are expressed as the mean ± SD (*n* = 8), evaluated using one-way ANOVA. ^#^*p* < 0.05, ^##^*p* < 0.01, compared with the normal group. **p < 0.01, compared with the model group.

Figure 2 shows that serum cortisol, total cholesterol, HDL-C and LDL-C in each group showed no significant difference. The serum testosterone levels and serum testosterone/cortisol (T/C) ratio in the model group (115.52 ± 13.05 μg/dL; 67.83 ± 14.29) both decreased by 38% on average when compared with the normal group (187.05 ± 44.31 μg/dL; 108.93 ± 36.00), which had a notable significant difference (*p* < 0.01). Compared with the model group, the serum testosterone levels and T/C ratio in EFE group (206.83 ± 5.54 μg/dL; 119.53 ± 25.04), the low-dose BYR group (210.60 ± 5.08 μg/dL; 119.38 ± 13.02), and the high-dose BYR group (220.57 ± 14.71 μg/dL; 124.26 ± 14.79) were increased significantly (*p* < 0.01). This leads to the conclusion that BYR can promote serum testosterone expression and increase the T/C ratio to regulate the metabolism process of the body.

### BYR protects and repairs the cellular endoplasmic reticulum and mitochondrial structure in leydig cells of EILST rats

**Figure 3.**
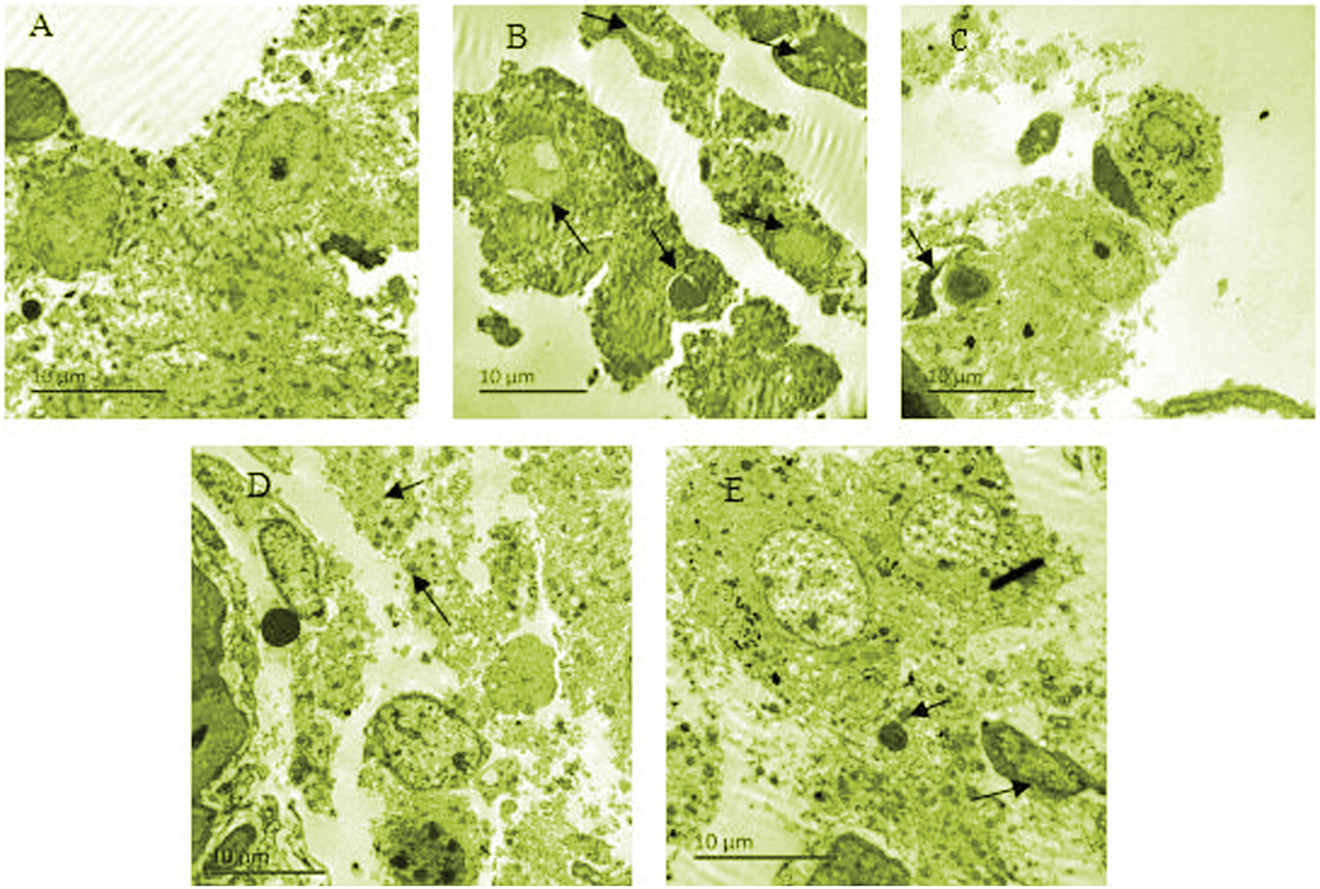
Ultrastructure of Leydig cells of rats of normal group (A), model group (B), arrowheads indicate many mitochondrial vacuoles were changed. *Epimedii folium* extract (EFE) group (C), arrowhead indicates ruptured cell membranes. Low-dose Bushen Yiyuan recipe (BYR) group (D), arrowheads indicate lysosomes increased. high-dose Bushen Yiyuan recipe (BYR) group (E), arrowheads indicate chromatin of some cells condensed in margin and lysosomes increased.

Figure 3 shows that the normal group had normal testicular cell structure, a few cells had slight edoema, but the structure of intracellular mitochondria and endoplasmic reticulum were clear. However, the Leydig cells of the model group showed severe interstitial edoema, endoplasmic reticulum expansion, and ruptured membranes. In few Leydig cells of this group, the mitochondrial vacuoles were changed, the condensed chromatin was in the margin and the nuclei were bare. The Leydig cells of the EFE group showed slight interstitial edoema, and ruptured cell membranes. In the low-dose BYR group, some Leydig cells had slight edoema, a few mitochondria vacuoles were changed, and lysosomes were increased. In the high-dose BYR group, it showed marginal condensed chromatin, visible cell structure, and endoplasmic reticulum. It was indicated that BYR can protect and repair the endoplasmic reticulum and mitochondrial structure in Leydig cells, maintain the morphology stability of testicular tissue.

### BYR significantly increased protein expression and mRNA expression level of HMG-CoA, STAR, CYP11A1 in EILST rats

**Figure 4.**
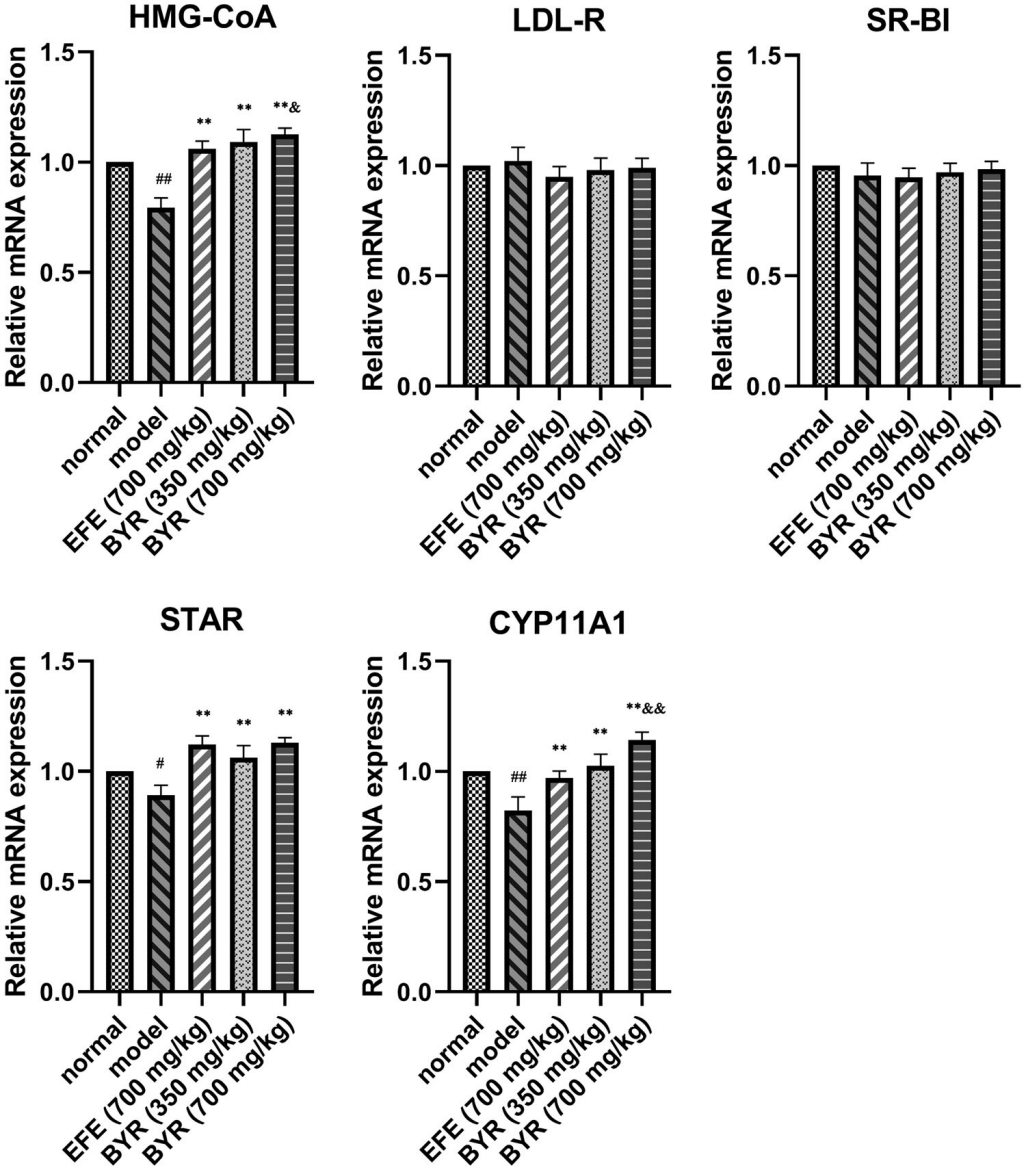
mRNA expression fold changes of HMG-CoA, LDL-R, SR-BI, STAR and CYP11A1 in rat Leydig cells of model group, *Epimedii folium* extract (EFE) group, low-dose Bushen Yiyuan recipe (BYR) group and high-dose Bushen Yiyuan recipe (BYR) group. Data are expressed as the mean ± SD (*n* = 8), evaluated using one-way ANOVA. ^#^*p* < 0.05, ^##^*p* < 0.01, compared with normal group. *p < 0.05, **p < 0.01, compared with model group. ^&^*p* < 0.05, ^&&^*p* < 0.01, compared with *Epimedii folium* extract (EFE) group.

**Figure 5.**
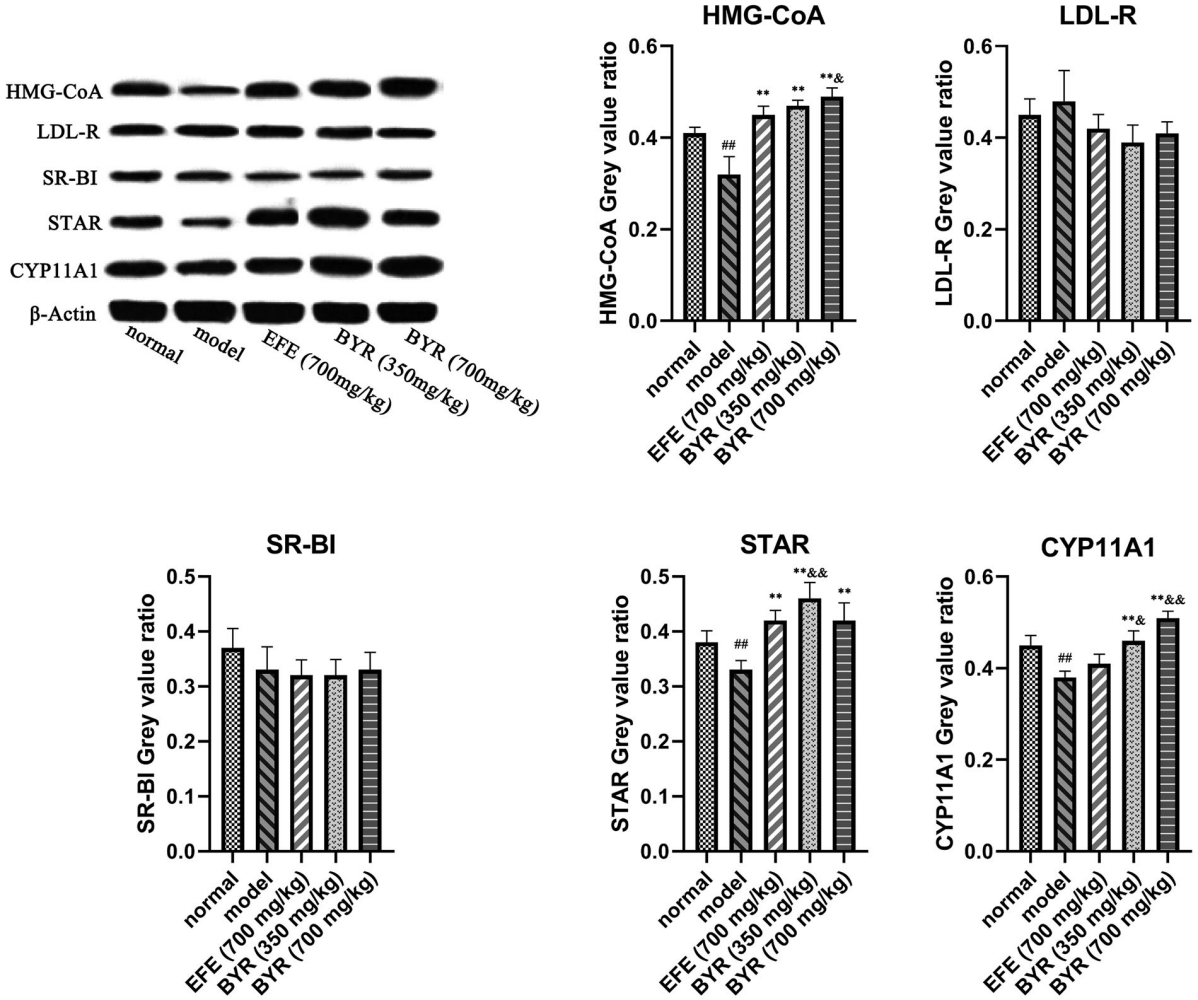
Protein expression of HMG-CoA, LDL-R, SR-BI, STAR and CYP11A1 in rat Leydig cells of model group, *Epimedii folium* extract (EFE) group, low-dose Bushen Yiyuan recipe (BYR) group and high-dose Bushen Yiyuan recipe (BYR) group. Data are expressed as the mean ± SD (*n* = 8), evaluated using one-way ANOVA. ^#^*p* < 0.05, ^##^*p* < 0.01, compared with the normal group. *p < 0.05, **p < 0.01, compared with the model group. ^&^*p* < 0.05, ^&&^*p* < 0.01, compared with *Epimedii folium* extract (EFE) group.

As shown in Figure 4, the relative expression of HMG-CoA, STAR and CYP11A1 mRNA in Leydig cells of rats in the model group (0.79 ± 0.05; 0.89 ± 0.45; 0.82 ± 0.06) was significantly lower than those in rats of the normal group (*p* < 0.01 or *p* < 0.05). In contrast to the model group, EFE and BYR at all doses remarkably promoted the relative expression level of HMG-CoA mRNA (1.06 ± 0.04; 1.09 ± 0.06; 1.13 ± 0.03), STAR mRNA (1.12 ± 0.04; 1.06 ± 0.06; 1.13 ± 0.02) and CYP11A1 mRNA (0.97 ± 0.03; 1.03 ± 0.05; 1.14 ± 0.04) (*p* < 0.01). The relative expression of HMG-CoA mRNA and CYP11A1 mRNA in high-dose BYR group significantly higher than those in rats of the EFE group (HMG-CoA mRNA, *p* < 0.05; CYP11A1 mRNA, *p* < 0.01). However, the relative expression of LDL-R and SR-BI mRNA showed no significant difference among each group. As shown in Figure 5, the expression of HMG-CoA, STAR and CYP11A1 proteins in Leydig cells of rats in the model group (0.32 ± 0.04; 0.33 ± 0.02; 0.38 ± 0.01) was significantly lower than those in rats of the normal group (0.41 ± 0.01; 0.38 ± 0.02; 0.45 ± 0.02) (*p* < 0.01). In contrast to the model group, BYR at low-dose and high-dose remarkably promoted the relative expression level of HMG-CoA protein (0.47 ± 0.01; 0.49 ± 0.02), STAR protein (0.46 ± 0.03; 0.42 ± 0.03) and CYP11A1 protein (0.46 ± 0.02; 0.51 ± 0.02) (*p* < 0.01). EFE can remarkably promoted the relative expression level of HMG-CoA protein (0.45 ± 0.02) (*p* < 0.01) and STAR protein (0.42 ± 0.02) (*p* < 0.01) but CYP11A1 protein (0.41 ± 0.02) (*p* > 0.05). The expression level of HMG-CoA and CYP11A1 proteins in high-dose BYR group significantly higher than those in rats of the EFE group (HMG-CoA protein, *p* < 0.05; CYP11A1 protein, *p* < 0.01), while relative expression level of STAR and CYP11A1 proteins in low-dose BYR group significantly higher than those in rats of the EFE group (STAR protein, *p* < 0.01; CYP11A1 protein, *p* < 0.05). However, there was no significant difference in the relative expression of LDL-R and SR-BI mRNA among each group. It was indicated that the BYR can treat EILST by promoting the expression of HMG-CoA, STAR and CYP11A1 protein.

## Discussion

EILST levels have been recognized as an urgent issue for athletes after a long period of high-intensity training. At present, pharmacological treatments such as testosterone or clomiphene citrate have been shown to be effective in treating EILST. However, these treatments are not available to athletes competing in sports governed by the World Anti-Doping Agency (Hooper et al. 2018). Traditional Chinese medicine has many advantages of being multi-component, multi-target, multi-pathway, and overall regulation of the body physiology (Wong et al. 2008). BYR, one of the safe and effective traditional Chinese medicine for the treatment of EILST, shows good prospects for development and utilization.

The mitochondria and endoplasmic reticulum play an important role in the synthesis and secretion of testosterone in testicular Leydig cells (Chen et al. 2019; Guan et al. 2018). Excessive apoptosis of testicular Leydig cells resulted in a significant decrease of testosterone secretion (Luo et al. 2011). We found that HIE damaged the ultrastructure of rat testicular Leydig cells, in particular, the model group showed edoema and necrosis of interstitial cells, the ruptured cell membrane, condensed chromatin in the margin, which would inevitably affect the synthesis and secretion of testosterone (Geniatulina et al. 2016). After dosing with BYR, the edoema condition was improved and the structures of endoplasmic reticulum, mitochondria and lysosomes were generally clearly seen, suggesting that the traditional Chinese medicine compound recipe BYR could maintain the function of testicular Leydig cells by protecting and repairing the endoplasmic reticulum and mitochondrial structures, thereby promoting the secretion of testosterone.

HMG-CoA, LDL-R, SR-BI, STAR and CYP11A1 are key enzymes in the synthesis process of testosterone in testicular Leydig cells (Liu et al. 2013). HMG-CoA reductase is a rate-limiting enzyme in the endogenous synthesis of cholesterol (Yu et al. 2015; Osaki et al. 2015; Yeganehjoo et al. 2017). HIE training causes the down-regulated expression of HMG-CoA reductase, which may be attributed to the decrease of serum testosterone levels caused by exercise (Cai et al. 2015). While serum testosterone was significantly increased in EFE, low-dose BYR and high-dose BYR groups, the expression of LDL-R mRNA was slightly reduced, suggesting that the biosynthesis of cholesterol in rats testicular Leydig cells that regulated by HMG-CoA reductase may be increased after drug dosing, which would inhibit the pathway of LDL-R transcription to intake exogenous cholesterol. In addition, compared with the normal group, SR-BI mRNA expression of the model, EFE, low-dose BYR and high-dose BYR groups were decreased, but there was no significant difference. However, the high-dose BYR group was closer to the normal group than the EFE and low-dose BYR group. Meanwhile, there was no significant change in total serum cholesterol, LDL-C, and HDL-C. Our study showed that SR-BI-mediated reverse uptake of exogenous cholesterol pathway was obstructed. Moreover, the changes in serum cholesterol may be related to the time, intensity, and the mode of exercise training.

Under normal physiological conditions, the first step of testosterone synthesis in Leydig cells is the rate-limiting step of STAR-mediated steroid synthesis. In this process, cholesterol is transported from mitochondria outer membrane into the mitochondrial inner membrane, and cholesterol on the inner surface of the matrix membrane of mitochondria is catalyzed to pregnenolone, the precursor of testosterone (Ojo et al. 2013; Luu-The 2013; Guan et al. 2017). The expression and activity of CYP11A1 and STAR were significantly correlated with testosterone production (Cunningham et al. 2016; Hatano et al. 2016) and inhibition of the expression of CYP11A1 and STAR has been reported to weaken testosterone production in Leydig cells (Guan et al. 2012; Guan et al. 2017; Lin et al. 2018). Our study showed that the mRNA expression of CYP11A1 and STAR in Leydig cells of testis was lower in the model group than in other groups, indicating that the expression of CYP11A1 and STAR was positively correlated with the testosterone production. Moreover, the highest expression level of CYP11A1 and STAR mRNA was found in the low-dose BYR group, suggesting that BYR could promote the synthesis process from cholesterol to testosterone. However, the specific active ingredients of BYR and the action mechanism of cholesterol synthesis affected by LDL-R transcription and/or SR-BI still need further study.

## Conclusions

BYR can affect serum testosterone levels through modulation of endogenous cholesterol synthesis and testosterone synthesis in testicular Leydig cells of rats with exercise- induced low serum testosterone levels. BYR remarkably promoted testosterone synthesis, the expression of HMG-CoA, STAR and CYP11A1 at protein and mRNA level, and increased testosterone/cortisol ratio. Additionally, BYR can protect and repair the endoplasmic reticulum and mitochondrial structure in Leydig cells, maintain the morphology stability of testicular tissue, and also contribute to testosterone secretion through promoting cholesterol biosynthesis and the transfer of cholesterol to the inner membrane of the mitochondria. The efficacy of BYR in the high-dose group is superior than that in the low-dose group and the positive control group of *Epimedii folium* extract. These results provide a possible theoretical basis for the clinical development of new sports nutrition supplements.
